# Impairments in psychological functioning in refugees and asylum seekers

**DOI:** 10.3389/fpsyg.2023.1295031

**Published:** 2024-01-08

**Authors:** Josef S. Baumgartner, Antonia Renner, Thomas Wochele-Thoma, Peter Wehle, Corrado Barbui, Marianna Purgato, Federico Tedeschi, Lorenzo Tarsitani, Valentina Roselli, Ceren Acartürk, Ersin Uygun, Minna Anttila, Tella Lantta, Maritta Välimäki, Rachel Churchill, Lauren Walker, Marit Sijbrandij, Pim Cuijpers, Markus Koesters, Thomas Klein, Ross G. White, Marion C. Aichberger, Johannes Wancata

**Affiliations:** ^1^Clinical Division of Social Psychiatry, Department of Psychiatry and Psychotherapy, Medical University of Vienna, Vienna, Austria; ^2^LBI Digital Health and Patient Safety, Vienna, Austria; ^3^Psychosocial Services in Vienna, Vienna, Austria; ^4^WHO Collaborating Centre for Research and Training in Mental Health and Service Evaluation, Department of Neurosciences, Biomedicine and Movement Sciences, Section of Psychiatry, University of Verona, Verona, Italy; ^5^Department of Human Neurosciences, Sapienza University of Rome, Rome, Italy; ^6^Department of Psychology, College of Social Sciences and Humanities, Koc University, Istanbul, Türkiye; ^7^Emergency and Disaster Management, Vocational School of Health Services, Bilgi University, Istanbul, Türkiye; ^8^Department of Nursing Science, Faculty of Medicine, University of Turku, Turku, Finland; ^9^Department of Public Health, Faculty of Medicine, University of Helsinki, Helsinki, Finland; ^10^Centre for Reviews and Dissemination, Faculty of Social Sciences, University of York, York, United Kingdom; ^11^School of Health and Social Work, University of Hertfordshire, Hatfield, United Kingdom; ^12^Department of Clinical, Neuro, and Developmental Psychology, Vrije Universiteit Amsterdam, Amsterdam, Netherlands; ^13^WHO Collaborating Centre for Research and Dissemination of Psychological Interventions, Vrije Universiteit Amsterdam, Amsterdam, Netherlands; ^14^Department of Psychiatry and Psychotherapy ll, District Hospital Guenzburg, Ulm University, Ulm, Germany; ^15^Center for Evidence-Based Healthcare, Medical Faculty Carl Gustav Carus, Technische Universität, Dresden, Germany; ^16^Institute of Population Health, Faculty of Health and Life Sciences, University of Liverpool, Liverpool, United Kingdom

**Keywords:** psychological functioning, refugee mental health, post-migration stressors, trauma, WHODAS 2.0, participation

## Abstract

Refugees are at increased risk for developing psychological impairments due to stressors in the pre-, peri- and post-migration periods. There is limited knowledge on how everyday functioning is affected by migration experience. In a secondary analysis of a study in a sample of refugees and asylum seekers, it was examined how aspects of psychological functioning were differentially affected. 1,101 eligible refugees and asylum seekers in Europe and Türkiye were included in a cross-sectional analysis. Gender, age, education, number of relatives and children living nearby, as well as indicators for depressive and posttraumatic symptoms, quality of life, psychological well-being and functioning, and lifetime potentially traumatic events were assessed. Correlations and multiple regression models with World Health Organization Disability Assessment Schedule 2.0 (WHODAS 2.0) 12-item version’s total and six subdomains’ scores (‘mobility’, ‘life activities’, ‘cognition’, ‘participation’, ‘self-care’, ‘getting along’) as dependent variables were calculated. Tests for multicollinearity and Bonferroni correction were applied. Participants reported highest levels of impairment in ‘mobility’ and ‘participation’, followed by ‘life activities’ and ‘cognition’. Depression and posttraumatic symptoms were independently associated with overall psychological functioning and all subdomains. History of violence and abuse seemed to predict higher impairment in ‘participation’, while past events of being close to death were associated with fewer issues with ‘self-care’. Impairment in psychological functioning in asylum seekers and refugees was related to current psychological symptoms. Mobility and participation issues may explain difficulties arising after resettlement in integration and exchange with host communities in new contexts.

## 1 Introduction

Everyday functioning is an essential part of one’s identity and is closely linked to quality of life and general well-being (Doré and Caron, 2017). Contrary to earlier conceptualisations of physical and mental health as the absence of illness, modern frameworks try to consider an individual’s abilities in light of its challenges and resources (Sleijpen et al., 2013). The World Health Organization (WHO) developed the International Classification of Functioning, Disability and Health (ICF) as a tool to describe information on functioning and to measure impairments in functioning in consideration of environmental and societal factors (World Health Organization, 2001). In accordance with the functioning levels of ICF, the WHO distributed the WHO Disability Assessment Schedule (WHODAS) version 2.0 to efficiently assess psychological functioning in general and clinical populations across the globe (Üstün et al., 2010). Psychological functioning is an umbrella term for the ability to interact with one’s surroundings and in different contexts.

Refugees and asylum seekers (RAS) face various pre-, peri- and post-migratory challenges (Laban et al., 2005; Priebe et al., 2016). RAS are often subjected to persecution for political, ethnic, religious or other reasons in their home countries, some witness death, combat, torture, abuse of relatives, or economic hardship and lack of water, food, and other basic needs (Nosè et al., 2020). On hazardous flight routes RAS are frequently exposed to physical harm, sexual violence and life-threatening conditions (Li et al., 2016). Even after resettlement in their host countries continuous stressors remain, including uncertainty about legal status, risk of being detained and deported, and difficulties with social integration, social exclusion, discrimination and economic disadvantages (Laban et al., 2008; Morgan et al., 2017). In a qualitative study, Salvo and de C Williams (2017) investigated how learning the host country’s language affected the lives of RAS. While the impact of learning the language was associated with achievement, aspirations and autonomy, barriers to learning, and a sense of shame due to reduced skills were important mitigators of individual well-being (Salvo and de C Williams, 2017). Furthermore, traumatic life events are associated with common mental disorders such as posttraumatic stress disorder (PTSD), and with reduced functioning, with a more pronounced relationship in women than in men (Robertson et al., 2016). Recent studies confirm the risk of psychological distress, and occurrence of depression and anxiety symptoms in RAS individuals from Ukraine (Buchcik et al., 2023), while identifying several coping and resilience strategies in the context of war as well as risk and protective factors (Oviedo et al., 2022; Rizzi et al., 2023).

However, outcomes may also be very dependent on the different modes of migration, reasons for flight, levels of openness or hostility of the host country society, as well as the legal, socioeconomic and healthcare provisions available in the host countries (Tay et al., 2019).

The aim of this cross-sectional analysis is to contribute to the existing knowledge base relating to risk factors associated with reduced psychological functioning overall and in the six domains of cognition, mobility, self-care, getting along, life activities and participation according to WHODAS 2.0 12-item version in a vulnerable, but clinically healthy population of RAS in high- and middle-income countries. To the best of our knowledge, no studies have yet examined influence factors on the different domains of functioning among RAS. Of particular interest will be which domains of functioning are typically impaired in these populations, whether specific effects of migration- and flight-associated stressors can be identified, and the specific role of traumatic events. Initial expectations were that, even in the absence of overt mental disorders, reduced psychological functioning would still play an important role in refugee mental health.

## 2 Method

### 2.1 Study design

We performed a secondary analysis of the RE-DEFINE dataset (Purgato et al., 2019, 2021; Acarturk et al., 2022), specifically of baseline data from an intervention study which had been gathered in the period between September 2018 and March 2020.

### 2.2 Participants and procedures

Recruitments took place in Austria, Finland, Germany, Italy, Türkiye and the United Kingdom. The study population had been highly selected, with inclusion and exclusion criteria rigorously applied in order to test the preventive effects of a psychosocial intervention in a sample of RAS who were in psychological distress, but without diagnosis of a psychiatric disorder. Inclusion criteria were (1) age 18 or above, (2) able to speak and understand Arabic, Dari, or English, (3) being asylum seeker, refugee or person under temporary protection, (4) presence of psychological distress with a score of 3 or more (binary scoring) on the 12-item General Health Questionnaire (GHQ-12), and (5) giving oral and written consent; exclusion criteria were (1) any mental disorder as shown by a positive Mini International Neuropsychiatric Interview (M.I.N.I., Sheehan et al., 1997), (2) acute medical conditions contraindicating participation, (3) clinical evidence of imminent suicide risk or `moderate or high` suicide risk on the M.I.N.I., and (4) clinical evidence of impaired decision-making, and are given also in the RE-DEFINE study protocols (Purgato et al., 2019). Interviews were performed with the help of cultural mediators and involved translators where necessary. Psychological distress, PTSD symptoms, depressive symptoms, psychological well-being and potentially traumatic life events were assessed by using standardized, widely used and feasible measures (see ‘2.3. Measures’). Furthermore, sociodemographic variables (age, gender, years and level of education, number of relatives and children living in the same country) and migration-associated factors (detention, post-migration living difficulties) were gathered during the interviews.

### 2.3 Measures

#### 2.3.1 WHODAS 2.0 12-item version

The World Health Organization Disability Assessment Schedule (WHODAS) 2.0 12-item version (short form) is a measurement for deficits in everyday functioning due to health conditions across six domains (Üstün et al., 2010). It is linked to the concepts of the International Classification of Functioning (World Health Organization, 2001) and assesses a time period of 30 days before the interview. The schedule, which was used in translated and validated versions, has shown excellent internal consistency with Cronbach’s alpha up to 0.96, and has been used across different countries and cultures with good discriminative ability and test–retest reliability (Saltychev et al., 2021). Two items each are grouped into the six domains ‘cognition’ (learning a new task, concentrating on doing something for 10 min), ‘mobility’ (standing for long periods, walking a long distance), ‘self-care’ (washing whole body, getting dressed), ‘getting along’ (dealing with people you do not know, maintaining friendships), ‘life activities’ (household responsibilities, day-to-day work), and ‘participation’ (joining community activities, emotionally affected by health problems). In our sample, Cronbach’s alpha for the WHODAS 2.0 was 0.84, and ranging from 0.41 (‘participation’) to 0.90 (‘self-care’) for the subdomains.

#### 2.3.2 PCL-5

The Posttraumatic Symptom Checklist (PCL-5) is based on the Diagnostic and Statistical Manual of Mental Disorders 5th edition (DSM-5)‘s criteria for PTSD (American Psychiatric Association, 2013; Blevins et al., 2015). PCL-5 comprises 20 items out of the diagnostic criteria domains ‘reexperiencing’, ‘avoidance’, ‘negative alterations in cognitions and mood’ and ‘hyperarousal’, and rates them on a scale from zero (‘not at all’) to four (‘extremely’). Recommended cut-offs for probable PTSD diagnosis and indication for treatment range from scores of 31–33 points (Bovin et al., 2016). Internal consistency was measured with a Cronbach’s alpha = 0.91 in this study.

#### 2.3.3 PHQ-9

The Patient Health Questionnaire 9-item version (PHQ-9) is a short screening tool for depressive symptoms in adult populations (Kroenke et al., 2001). It rates the nine Diagnostic and Statistical Manual of Mental Disorders 4th edition (DSM-IV)‘s criteria for depression from zero (‘not at all’) to three (‘nearly every day’) (American Psychiatric Association, 1994). The PHQ-9 is a useful tool for measuring depression, with a recommended cut-off of 10 points, and depression severity, with scores of 5, 10, 15, and 20 indicating mild, moderate, moderately severe, and severe depressive symptoms, respectively (Kroenke et al., 2001). Cronbach’s alpha was 0.83 in this study.

#### 2.3.4 GHQ-12

The General Health Questionnaire 12-item version (GHQ-12) is a short reliable measurement for psychological distress and signs of depression and anxiety (Goldberg et al., 1997). It includes a four-point Likert scale on each of the 12 items, and is most commonly rated either in a 0–0–1-1 or 0–1–2-3 mode. In order to better assess for severity of distress the second mode was used, with a Cronbach’s alpha of 0.69.

#### 2.3.5 PMLD

Stressors due to current living conditions in the host country were assessed through 17 items of the Post-Migration Living Difficulties (PMLD) scale, which rates if people were affected by problems in various domains from zero (‘not a problem’) to four (‘a very serious problem’) in the past 12 months (Silove et al., 1997). Cronbach’s alpha in our sample was 0.85.

#### 2.3.6 WHO-5

The 5-item World Health Organization Well-Being Index (WHO-5) is a commonly used short assessment of psychological wellbeing (World Health Organization, Regional Office, 1998). Five positively framed questions on subjective well-being are scored from zero (‘at no time’) to five (‘all of the time’), and generated scores from zero to 25 are elsewhere usually multiplied by four (0–100 final score). It has been applied in many languages and across different settings, and has shown adequate validity as an outcome measure (Cronbach’s alpha in this sample 0.88), with scores of 50 or below indicating poor well-being (12.5 in this study), and as a screening tool for depression (cut-off 28; or 7 in our study) (Topp et al., 2015).

#### 2.3.7 HTQ part I

The Harvard Trauma Questionnaire (HTQ) assesses posttraumatic symptoms and life-time traumatic events in refugees (Mollica et al., 1992). Its first part focuses on 16 specific traumatic events and one item ‘other (e.g., domestic violence)’, and had been assessed in a binary yes or no question in this study. In a principal component analysis including a larger sample of refugees and asylum seekers HTQ items had been pooled in three thematic domains ‘lack of basic needs’ (three items, ‘factor 1’), ‘violence and abuse’ (eight items, ‘factor 2’) and ‘being close to death’ (six items, ‘factor 3’) (Barbui et al., 2023). Internal consistency was high with Cronbach’s alpha of 0.87 (subscores’ alphas 0.75–0.80).

### 2.4 Data analysis

Sociodemographic, migration-related factors and results of WHODAS 2.0, GHQ-12, HTQ-I, PCL-5, PHQ-9, PMLD and WHO-5 were analyzed by using descriptive statistics, in absolute numbers and percentages for the categorical variables, and with means and standard deviations for continuous parameters. All parameters are given in gender-disaggregated form in order to take into account gender differences in descriptive statistics. Cross-tabulation for the WHODAS 2.0 subdomains was used to describe the level of functioning in the sample, with mean and standard deviation (SD) at the domain level to sense differences in impairment. Overall WHODAS 2.0 scores were also reported, to see if values are evenly distributed in our sample.

Spearman rank-order correlations between WHODAS 2.0 total scores (0–48) and each domain score separately (0–8) were calculated for all potential factors, in order to detect strong positive or negative relationships. Strengths of correlations were measured with Spearman correlation coefficients r_s_.

Finally, multivariable linear regression models were performed for WHODAS 2.0 scores and sub-scores separately, with all other variables acting simultaneously as independent variables (SPSS ‘Enter’ command). Non-standardized regression coefficients were calculated, and variance inflation factor (VIF) as a test for multi-collinearity was applied. In order to account for multiple testing, Bonferroni correction (x7) was used, and the significance level of *p* < 0.05 was adjusted accordingly (*p* < 0.007).

## 3 Results

Sociodemographic characteristics are summarized in Table 1. A total of 1,101 RAS (48.9% female) with a mean age of 31.8 years (SD 9.5; range 18–71 years) were included in the analysis, the majority of which were of Syrian nationality (*n* = 758; 68.8%) and resettled to Türkiye (*n* = 642; 58.3%). Male RAS reported considerably more often having been held in detention than female RAS (24.2% vs. 6.3%), were less likely to be living in a marriage or partnership (57.0% vs. 77.7%), and had on average fewer children and relatives living in the close proximity or the same household. Otherwise, men and women were roughly comparable in other sociodemographic variables.

**Table 1:** Sociodemographics of total sample (*n* = 1,101).

Sociodemographic variable	Male (*n* = 563)	Female (*n* = 538)
Age in years, mean (SD)	31.6 (±9.2)	32.0 (±9.9)
Education in years, mean (SD)	9.8 (±4.5)	9.3 (±4.3)
Education level, n (%)		
Illiterate	31 (5.5%)	43 (8.0%)
Primary school	300 (53.3%)	291 (54.1%)
High school	126 (22.4%)	117 (21.7%)
University	98 (17.4%)	87 (16.2%)
Country of origin, n (%)		
Afghanistan	39 (6.9%)	27 (5.0%)
Iraq	66 (11.7%)	28 (5.2%)
Nigeria	92 (16.3%)	22 (4.1%)
Pakistan	41 (7.3%)	0 (0.0%)
Syria	311 (55.2%)	447 (83.1%)
Other/unknown	14 (2.5%)	14 (2.6%)
Host country, n (%)		
Austria	39 (6.9%)	27 (5.0%)
Finland	74 (13.1%)	26 (4.8%)
Germany	27 (4.8%)	15 (2.8%)
Italy	137 (24.3%)	22 (4.1%)
Türkiye	238 (42.3%)	404 (75.1%)
United Kingdom	48 (8.5%)	44 (8.2%)
Relationship status, n (%)		
Single	229 (40.7%)	77 (14.3%)
married-partnership	321 (57.0%)	418 (77.7%)
divorced	10 (1.8%)	27 (5.0%)
widowed	1 (0.1%)	15 (2.8%)
Number of relatives, mean (SD)	2.5 (±2.7)	4.6 (±3.4)
Number of children, mean (SD)	1.7 (±2.2)	2.6 (±2.0)
Unemployed, n (%)	272 (48.3%)	147 (27.3%)
Detention during migration, n (%)	136 (24.2%)	34 (6.3%)

SD, standard deviation.

Presence of psychiatric disorder as assessed by the M.I.N.I. was one of the exclusion criteria, but, based on a GHQ score of 3 or above, those assessed as experiencing clinically significant distress were eligible, and data on a range of depressive symptoms and PTSD symptoms was collected (see Table 2). Men suffered on average more post-migration living difficulties, and reported more lifetime potentially traumatic events, especially those associated with violence and close-to-death experiences. Participants in general showed high psychological distress and reduced quality of life. Accordingly, as shown in Table 3, everyday functioning was mild to moderately impaired in the cohort. The most strongly affected domains of functioning were ‘mobility’ and ‘participation’, followed by ‘life activities’ and ‘cognition’.

**Table 2:** Depressive and PTSD symptoms, traumatic events, post-migration living difficulties, quality of life and psychological problems in the sample.

Psychometric measure	Male (*n* = 563)	Female (*n* = 538)
PHQ-9 total score (0–27), mean (SD)	7.1 ( ± 5.2)	7.3 ( ± 5.2)
PCL-5 total score (0–80), mean (SD)	20.9 ( ± 15.4)	22.6 ( ± 15.3)
HTQ part I (0–17), mean (SD)	6.9 ( ± 4.4)	4.7 ( ± 4.0)
factor 1: lack of basic needs (0–3), mean (SD)	1.5 ( ± 1.2)	1.1 ( ± 1.1)
factor 2: violence and abuse (0–8), mean (SD)	2.3 ( ± 2.2)	1.2 ( ± 1.8)
factor 3: being close to death (0–6), mean (SD)	3.1 ( ± 1.9)	2.4 ( ± 1.9)
PMLD (0–68), mean (SD)	22.9 ( ± 13.0)	18.3 ( ± 11.8)
WHO-5 total (0–25), mean (SD)	11.7 ( ± 6.1)	10.6 ( ± 6.0)
GHQ-12 (0–36), mean (SD)	16.6 ( ± 4.4)	17.4 ( ± 4.4)

DSM-5, Diagnostic and Statistical Manual of Mental Disorders 5th edition; GHQ-12, General Health Questionnaire 12-item version; HTQ, Harvard Trauma Questionnaire; PCL-5, PTSD Checklist for DSM-5; PHQ-9, Patient Health Questionnaire 9-item version; PMLD, post migration living difficulties form; PTSD, Post-traumatic Stress Disorder; SD, standard deviation; WHO-5, 5-item World Health Organization Well-Being Index.

**Table 3:** 12-item WHODAS 2.0 total and domain scores in the sample.

WHODAS 2.0 score	Male (*n* = 563)	Female (*n* = 538)
WHODAS 2.0 total score (0–48), mean (SD)	6.1 ( ± 6.5)	7.1 ( ± 7.5)
WHODAS 2.0 domain mobility (0–8), mean (SD)	1.2 ( ± 1.9)	1.8 ( ± 2.3)
WHODAS 2.0 domain life activities (0–8), mean (SD)	1.3 ( ± 1.7)	1.4 ( ± 1.8)
WHODAS 2.0 domain cognition (0–8), mean (SD)	1.2 ( ± 1.6)	1.3 ( ± 1.8)
WHODAS 2.0 domain participation (0–8), mean (SD)	1.5 ( ± 1.6)	1.4 ( ± 1.8)
WHODAS 2.0 domain selfcare (0–8), mean (SD)	0.2 ( ± 1.0)	0.3 ( ± 1.1)
WHODAS 2.0 domain getting along (0–8), mean (SD)	0.7 ( ± 1.4)	0.8 ( ± 1.6)

SD, standard deviation; WHODAS 2.0, World Health Organization Disability Assessment Schedule 2.0.

Spearman rank-order correlations revealed that reduced overall psychological functioning was moderately to strongly associated with PHQ-9 depressive (r_s_ = 0.49) and PCL-5 PTSD (r_s_ = 0.55) symptom scores. Moderate correlations with depressive (PHQ-9) and PTSD (PCL-5) symptoms were also seen for the WHODAS 2.0 domains ‘life activities’ (r_s_ = 0.37 and 0.41), ‘cognition’ (r_s_ = 0.40 and 0.46), ‘participation’ (r_s_ = 0.45 and 0.46), and ‘getting along’ (r_s_ = 0.31 and 0.34). PHQ-9 and PCL-5 scores correlated strongly with each other (r_s_ = 0.68), and both had an inverse relationship to quality of life (r_s_ = 0.41 and 0.34). All bivariate correlations are shown in a table in the appendix (Supplementary Table S1).

The fitted regression models for WHODAS 2.0 total score and domain scores are shown in Table 4. Overall regressions were statistically significant (all *p* < 0.001), with a variance explained by all predictors simultaneously of around one-third (Nagelkerke’s *R*^2^ = 0.368). It was found that older age was significantly associated with more pronounced disability overall (*p* < 0.001) as well as in the specific domains ‘mobility’ (*p* < 0.001) and ‘participation’ (*p* = 0.001). Higher PCL-5 and PHQ-9 scores were associated with worse psychological functioning overall (both *p* < 0.001) and in every subdomain (all *p* < 0.001, except PHQ-9 with ‘mobility’ (*p* = 0.004)). Post-migration living difficulties was associated with worse functioning in ‘cognition’ (*p* = 0.003). Traumatic life events seemed to have heterogeneous effects on psychological functioning. While ‘violence and abuse’ (HTQ I factor 2) was significantly associated with impairment in ‘participation’ (*p* = 0.007), ‘being close to death’ (HTQ I factor 3) was non-significantly associated to lower disability overall (*p* = 0.042), and better ‘self-care’ significantly (*p* < 0.001). Worse ‘self-care’, on the other hand, was significantly associated with a higher number of children (*p* < 0.001). The highest VIF was tolerable (VIF = 2.177), hence supporting the assumption of non-multicollinearity of parameters.

**Table 4:** Multiple linear regression analyses with WHODAS 2.0 total score and subdomain scores as dependent variables, with non-standardized regression coefficients B for each independent variable, number of observations, R and F statistics and VIF.

VARIABLE	WHODAS 2.0 total score	WHODAS 2.0 mobility	WHODAS 2.0 life activities	WHODAS 2.0 cognition	WHODAS 2.0 participation	WHODAS 2.0 self-care	WHODAS 2.0 getting along
Constant	−2.618*	−1.196*	−0.441	−0.275	−0.481	−0.202	−0.022
Age	0.077*	0.037*	0.011	0.011	0.020*	−0.005	0.003
Female gender	−0.096	0.219	−0.123	−0.058	−0.141	−0.016	0.019
Education level	−0.071	−0.127	0.061	−0.160	0.038	0.029	0.087
Number of relatives	0.029	0.032	0.064	−0.005	−0.010	−0.027	−0.024
Number of children	0.236	0.118	0.013	0.018	−0.040	0.145*	−0.019
Detention	0.269	0.192	−0.054	0.168	0.037	0.118	−0.193
PCL-5	0.169*	0.038*	0.029*	0.039*	0.033*	0.010*	0.020*
PHQ-9	0.343*	0.053*	0.067*	0.064*	0.072*	0.029*	0.058*
PMLD	0.030	0.005	0.004	0.013*	0.007	0.002	−0.001
HTQ I ‘lack of basic needs’ (factor 1)	0.061	0.105	−0.044	0.025	−0.055	0.043	−0.013
HTQ I ‘violence and abuse’ (factor 2)	0.033	−0.063	<0.001	−0.021	0.094*	−0.005	0.029
HTQ I ‘being close to death’ (factor 3)	−0.253	−0.046	0.001	−0.051	−0.021	−0.069*	−0.068
Observations	770	770	770	770	770	769	770
R^2^	0.378	0.221	0.183	0.293	0.290	0.180	0.158
Adjusted R^2^	0.368	0.209	0.171	0.281	0.279	0.167	0.145
Residual standard error	5.36198	1.84901	1.58955	1.39729	1.42703	0.85536	1.31349
F statistics (df = 12)	38.315*	17.902*	14.176*	26.079*	25.768*	13.831*	11.841*
Highest VIF (HTQ I factor 2 in all regression models)	2.174	2.174	2.174	2.174	2.174	2.177	2.174

df, degrees of freedom; DSM-5, Diagnostic and Statistical Manual of Mental Disorders 5th edition; HTQ, Harvard Trauma Questionnaire; PCL-5, PTSD Checklist for DSM-5; PHQ-9, Patient Health Questionnaire 9-item version; PMLD, post migration living difficulties form; PTSD, Post-traumatic Stress Disorder; VIF, variance inflation factor; WHODAS 2.0, World Health Organization Disability Assessment Schedule 2.0.

* *p* < 0.007 (corresponds to 0.05, after Bonferroni correction).

## 4 Discussion

This study describes deficits in psychological functioning among a large sample of RAS in high- and middle-income countries in Europe and Türkiye. Functioning is more commonly investigated as a secondary or control variable, but rarely considered as an important primary outcome. It adds value to scientific knowledge in the sense of a differential analysis of psychological functioning in a predominantly Arabic-speaking sample of RAS in European countries including Türkiye. Syria is currently the main origin of RAS worldwide, who are experiencing a high prevalence of mental disorders (Sá et al., 2022), and together with RAS from Ukraine and Afghanistan account for 52% of refugees and persons needing protection worldwide in 2022 (UNHCR, 2023).

### 4.1 Impairments in psychological functioning

An individual’s everyday functioning - i.e. coping with everyday stressors and mastering challenges - is a multi-faceted ability, and there are calls for assessing different domains of functioning rather than an overall functioning as a summative score (Saltychev et al., 2021). These findings support our initial expectation that the various domains of functioning are differentially influenced by pre-, peri- and post-migration parameters. Our sample of RAS exhibits notable impairments in functioning, as the mean overall WHODAS 2.0 scores in the sample lie between the 80^th^ and 90^th^ percentile of population percentiles of the 12-item version (Ustun et al., 2012). Consistent with findings from other studies of RAS, older age is associated with disability, which might be due to the effects of physical aging (Mollica, 1999).

In particular, symptoms of depression and PTSD were linked to worse day-to-day functioning overall and in all functioning subdomains in RAS with psychological distress. The influence of potentially traumatic life events, which correlated poorly with PTSD symptom scores, is more complex. In this study, experiences of deprivation and of violence and abuse were associated with lower functioning, especially when people were asked if they had trouble in joining community activities and if they were emotionally affected by their health problems. Counter-intuitively, experiences of ‘being close to death’, e.g., witnessing murder/combat, were associated with lower WHODAS 2.0 scores, and significantly predicted better functioning in ‘self-care’ (personal hygiene and dressing oneself). A similar result has been found in people who were victims of torture, but with a very small impact (Laban et al., 2008). In this population of non-hospitalized RAS individuals, these results might be explained by a higher level of resilience in those who have managed to cope with such difficulties. Similarly, front line workers in the COVID-19 pandemic who exhibited high trait resilience were tolerating stressors better (Fino et al., 2021). Another mechanism could be seen in accounts of post-traumatic growth (PTG) after traumatic events, where individuals were able to maintain or even exceed their functioning facing adversity (Tedeschi and Calhoun, 2004). For refugees, evidence of PTG was reported in several studies (Sultani et al., 2024).

Generally, it is acknowledged that trauma is a critical harmful agent in many aspects of everyday functioning, which is well documented for different populations of RAS (Khan and Haque, 2021). These findings underline the necessity of recognizing that trauma effects on individual functioning may be multifactorial, and of always considering multiple influencing factors, such as symptoms of PTSD, family support and living conditions (Mollica, 1999). Disability in getting acquainted with living conditions and new people can maintain and even reinforce disadvantages, with consequences of prolonged suffering from mental health disorders (Friis Jørgensen et al., 2017).

Difficulties with living conditions post-migration were associated with worse functioning in concentrating and learning new tasks. Structuring one’s daily routines and being resilient are important assets in organizing one’s own day-to-day life, and difficult living arrangements, such as over-crowding, limited privacy and conflicts in large communal accommodations, may negatively interfere with these (Agbih, 2019). Another finding is that a higher number of children in the same household or living nearby was associated with worse ‘self-care’ and with worse functioning overall, which was previously described (Robertson et al., 2016). On the one hand, this could be explained by limited time resources when caring for under-aged children, and therefore having less time for fulfilling one’s own needs, also considering that finding childcare options for RAS is difficult. On the other hand - with women reporting having more children than men in our sample – this could be a reflection of differences between genders, as the majority of RAS in Türkiye were female, whereas RAS in Western European countries were predominantly male. Another study reported a stronger negative relationship between trauma and functioning for females than for males, which might add to the gender differences (Robertson et al., 2016).

Lastly, the observed association between experiences of violence and abuse, and lower abilities in participating in community activities could stem from certain stressors or definitive life events. Past exposure to traumatic events may lead the individual to be less inclined to want to participate, potentially posing a further barrier toward inclusion into the host society, especially as expectations that RAS should integrate with the community in host countries may further amplify individual stress, potentially leading to social exclusion.

As one strategy to prevent social exclusion, proficiency courses in the host country’s language could be combined with work training, day structure training and opportunities to mingle with local communities. Combined language and work training programs show promising results (Kuschel et al., 2023). Isolated language tandem programs have not been scientifically evaluated in RAS, gender differences must be taken into consideration in these populations. Female RAS’s relationship status and childcare situations have an impact on their language skills, which was not found for male RAS (Bernhard and Bernhard, 2022). Having fewer opportunities to learn than men, female RAS were more efficient in learning (Bernhard and Bernhard, 2022).

### 4.2 Strengths and limitations

Strengths of this study are: firstly, a large sample size of RAS from many countries and settings: secondly, a methodologically exact screening procedure which rendered a comparably homogenous sample of RAS: thirdly, the nationalities included show that the sample was quite representative for current RAS populations in Europe and Türkiye until 2022. However, conclusions cannot be made for the general population of RAS in these regions, as we investigated a non-random sample under psychological distress and interested to receive an intervention. Limiting the study’s findings is foremost that the study was ideated for another type of analysis; in this sample, psychological functioning was primarily assessed as a secondary outcome. Secondly, the cross-sectional methodology does not warrant causality assumptions, and some associations could be explained by reverse causality. Thirdly, we did not assess physical health, which might have been an important predictor of psychological functioning in our sample. Fourthly, questionnaires derived from different versions of the DSM were used, which might limit their comparability. Generated hypotheses have to be reviewed in a longitudinal follow-up study.

### 4.3 Conclusion

This study underlines the need for the consideration of sub-clinical forms of war and trauma-associated disabilities, as patterns of functional impairment are even present in RAS without a clinical diagnosis of a mental health disorder. This finding might be taken into account in the organization of medical and psychosocial assistance to RAS. We hypothesize that reduced everyday functioning of individuals who had to leave their home country due to war, prosecution, and other hardships could hamper their ability to adapt successfully to new contexts, cope with new tasks and recover from their experiences.

## Data availability statement

The raw data supporting the conclusions of this article will be made available by the authors, without undue reservation.

## Ethics statement

The studies involving humans were approved by WHO Research Ethics Review Committee and Ethics Committees of all participating sites. The studies were conducted in accordance with the local legislation and institutional requirements. The participants provided their written informed consent to participate in this study.

## Author contributions

JB: Conceptualization, Data curation, Formal analysis, Investigation, Methodology, Writing – original draft, Writing – review & editing. AR: Writing – review & editing, Visualization TW-T: Writing – review & editing. PW: Writing – review & editing. CB: Conceptualization, Funding acquisition, Project administration, Supervision, Writing – review & editing. MP: Writing – review & editing. FT: Data curation, Formal analysis, Supervision, Writing – review & editing. LT: Writing – review & editing. VR: Writing – review & editing. CA: Writing – review & editing. EU: Writing – review & editing. MA: Writing – review & editing. TL: Writing – review & editing. MV: Writing – review & editing. RC: Writing – review & editing. LW: Writing – review & editing. MS: Writing – review & editing. PC: Writing – review & editing. MK: Writing – review & editing. TK: Writing – review & editing. RW: Writing – review & editing. MCA: Conceptualization, Supervision, Writing – review & editing, Methodology, Validation. JW: Writing – review & editing, Conceptualization, Project administration, Supervision.

## Supplementary material

Supplementary Table S1 Spearman correlations.
