# Supplementary material for: Impairments in psychological functioning in refugees and asylum seekers
Source: Front Psychol. 2024 Jan 8;14:1295031. doi: 10.3389/fpsyg.2023.1295031 (PMC10801113; doi:10.3389/fpsyg.2023.1295031)
Supplement: Supplementary Table S1 — Spearman correlations. [file Table_1.DOCX]

Supplementary material

TABLE S1: Spearman-Rho correlation coefficients of items at baseline and PMLD

|  | WHODAS 2.0 total | WHODAS 2.0 mobility | WHODAS 2.0 life activities | WHODAS 2.0 cognition | WHODAS 2.0 participation | WHODAS 2.0 self-care | WHODAS 2.0 getting along | Age | Female gender | Education level | Number of relatives | Number of children | Detention | GHQ-12 | HTQ I lack of needs | HTQ I violence and abuse | HTQ I being close to death | PCL-5 | PHQ-9 | PMLD | WHO-5 |
| --- | --- | --- | --- | --- | --- | --- | --- | --- | --- | --- | --- | --- | --- | --- | --- | --- | --- | --- | --- | --- | --- |
| WHODAS 2.0 total | 1 | .713 | .759 | .754 | .747 | .397 | .543 | .147 | .048 | -.032 | -.025 | .106 | .025 | .273 | .186 | .181 | .154 | .547 | .486 | .154 | -.288 |
| WHODAS 2.0 mobility |  | 1 | .474 | .446 | .381 | .313 | .240 | .201 | .129 | -.064 | .146 | .194 | -.069 | .272 | .083 | .015 | .042 | .358 | .292 | .058 | -.233 |
| WHODAS 2.0 life activities |  |  | 1 | .523 | .480 | .301 | .330 | .119 | .043 | .032 | .054 | .118 | -.037 | .180 | .084 | .110 | .101 | .405 | .371 | .072 | -.254 |
| WHODAS 2.0 cognition |  |  |  | 1 | .507 | .253 | .384 | .092 | .003 | -.081 | -.064 | .050 | .065 | .215 | .171 | .150 | .113 | .456 | .401 | .194 | -.179 |
| WHODAS 2.0 participation |  |  |  |  | 1 | .250 | .381 | .070 | -.048 | .017 | -.200 | -.042 | .107 | .210 | .216 | .281 | .198 | .458 | .447 | .191 | -.233 |
| WHODAS 2.0 self-care |  |  |  |  |  | 1 | .202 | .041 | .036 | -.011 | .055 | .156 | -.002 | .159 | .108 | .040 | -.016 | .254 | .210 | .038 | -.107 |
| WHODAS 2.0 getting along |  |  |  |  |  |  | 1 | .002 | .053 | .007 | -.091 | -.020 | -.005 | .177 | .107 | .105 | .075 | .338 | .311 | .052 | -.143 |
| Age |  |  |  |  |  |  |  | 1 | .007 | -.011 | .191 | .472 | -.147 | .004 | -.014 | -.076 | .074 | .030 | .014 | -.020 | -.016 |
| Female gender |  |  |  |  |  |  |  |  | 1 | -.037 | .365 | .268 | -.244 | .101 | -.180 | -.267 | -.175 | .068 | .025 | -.176 | -.090 |
| Education level |  |  |  |  |  |  |  |  |  | 1 | -.096 | -.132 | .001 | -.025 | -.048 | .037 | .081 | .033 | .024 | .040 | .037 |
| Number of relatives |  |  |  |  |  |  |  |  |  |  | 1 | .659 | -.358 | .002 | -.257 | -.440 | -.263 | -.079 | -.138 | -.291 | -.042 |
| Number of children |  |  |  |  |  |  |  |  |  |  |  | 1 | -.283 | .012 | -.115 | -.259 | -.126 | -.001 | -.060 | -.158 | -.052 |
| Detention |  |  |  |  |  |  |  |  |  |  |  |  | 1 | .006 | .267 | .384 | .184 | .020 | .069 | .207 | .025 |
| GHQ-12 |  |  |  |  |  |  |  |  |  |  |  |  |  | 1 | .115 | .073 | .107 | .348 | .388 | .103 | -.232 |
| HTQ I lack of needs |  |  |  |  |  |  |  |  |  |  |  |  |  |  | 1 | .530 | .468 | .243 | .244 | .267 | -.115 |
| HTQ I violence |  |  |  |  |  |  |  |  |  |  |  |  |  |  |  | 1 | .570 | .273 | .269 | .286 | -.096 |
| HTQ I being close to death |  |  |  |  |  |  |  |  |  |  |  |  |  |  |  |  | 1 | .230 | .239 | .206 | -.065 |
| PCL-5 |  |  |  |  |  |  |  |  |  |  |  |  |  |  |  |  |  | 1 | .677 | .183 | -.341 |
| PHQ-9 |  |  |  |  |  |  |  |  |  |  |  |  |  |  |  |  |  |  | 1 | .212 | -.413 |
| PMLD |  |  |  |  |  |  |  |  |  |  |  |  |  |  |  |  |  |  |  | 1 | -.116 |
| WHO-5 |  |  |  |  |  |  |  |  |  |  |  |  |  |  |  |  |  |  |  |  | 1 |

DSM-5=Diagnostic and Statistical Manual of Mental Disorders 5^th^ edition; GHQ-12=General Health Questionnaire 12-item version; HTQ=Harvard Trauma Questionnaire; PCL-5=PTSD Checklist for DSM-5; PHQ-9=Patient Health Questionnaire 9-item version; PMLD=post migration living difficulties form; PTSD=Post-traumatic Stress Disorder; WHODAS 2.0=World Health Organization Disability Assessment Schedule 2.0; WHO-5=5-item World Health Organization Well-Being Index
